# A Reliable Multifaceted Solution against Foodborne Viral Infections: The Case of RiLK1 Decapeptide

**DOI:** 10.3390/molecules29102305

**Published:** 2024-05-14

**Authors:** Emanuela Galatola, Bruna Agrillo, Marta Gogliettino, Gianna Palmieri, Serena Maccaroni, Teresa Vicenza, Yolande T. R. Proroga, Andrea Mancusi, Simona Di Pasquale, Elisabetta Suffredini, Loredana Cozzi

**Affiliations:** 1 Institute of Biosciences and BioResources (IBBR), National Research Council (CNR), 80131 Naples, Italy; emanuela.galatola@ibbr.cnr.it (E.G.); bruna.agrillo@ibbr.cnr.it (B.A.); marta.gogliettino@ibbr.cnr.it (M.G.); 2 Materias Srl, 80146 Naples, Italy; 3 National Reference Laboratory for Foodborne Viruses, Department of Food Safety, Nutrition and Veterinary Public Health, Istituto Superiore di Sanità, 00161 Rome, Italy; serenamaccaroni96@gmail.com (S.M.); teresa.vicenza@iss.it (T.V.); simona.dipasquale@iss.it (S.D.P.); elisabetta.suffredini@iss.it (E.S.); loredana.cozzi@iss.it (L.C.); 4 Department of Food Microbiology, Istituto Zooprofilattico Sperimentale del Mezzogiorno, 80055 Portici, Italy; yolande.proroga@izsmportici.it (Y.T.R.P.); andrea.mancusi@izsmportici.it (A.M.)

**Keywords:** antimicrobial peptide, enteroviruses, non-enveloped virus, virucidal activity, foodborne viral infections, hepatitis A virus, murine norovirus

## Abstract

Food-borne transmission is a recognized route for many viruses associated with gastrointestinal, hepatic, or neurological diseases. Therefore, it is essential to identify new bioactive compounds with broad-spectrum antiviral activity to exploit innovative solutions against these hazards. Recently, antimicrobial peptides (AMPs) have been recognized as promising antiviral agents. Indeed, while the antibacterial and antifungal effects of these molecules have been widely reported, their use as potential antiviral agents has not yet been fully investigated. Herein, the antiviral activity of previously identified or newly designed AMPs was evaluated against the non-enveloped RNA viruses, hepatitis A virus (HAV) and murine norovirus (MNV), a surrogate for human norovirus. Moreover, specific assays were performed to recognize at which stage of the viral infection cycle the peptides could function. The results showed that almost all peptides displayed virucidal effects, with about 90% of infectivity reduction in HAV or MNV. However, the decapeptide RiLK1 demonstrated, together with its antibacterial and antifungal properties, a notable reduction in viral infection for both HAV and MNV, possibly through direct interaction with viral particles causing their damage or hindering the recognition of cellular receptors. Hence, RiLK1 could represent a versatile antimicrobial agent effective against various foodborne pathogens including viruses, bacteria, and fungi.

## 1. Introduction

According to the World Health Organization (WHO), foodborne diseases are a major safety concern worldwide, affecting approximately 30% of the population in developed countries annually [1]. Each year, foodborne illnesses affect over 600 million people worldwide and result in over 420,000 deaths, 125,000 of which are in children [2].

Most food-related diseases are a result of microbial contamination, principally from bacteria, fungi, and viruses. Pathogens can be transferred to food products through contact with contaminated surfaces, especially with biofilms, which are more resistant to biocidal chemicals and conventional methods for microbial cleaning than free-living cells, as they are generally composed of multiple species showing a special physiology and physical matrix barrier [3,4,5,6]. As a result, preventing or eliminating contamination in food-processing environments, especially when ready-to-eat foods are handled, represents an arduous challenge. In this context, virus contamination, which is one of the important causes of foodborne illness worldwide, is recognized as a top priority among the microbiological food safety risks [7]. Specifically, norovirus (NoV) and hepatitis A virus (HAV) are considered to have a very relevant public health impact [8,9]. They are leading agents of acute gastroenteritis and hepatitis, respectively, due to their low infection dose, wide transmission route, strong environmental resistance, and ease of contaminating products such as fruit, lettuce, and shellfish, causing foodborne outbreaks [10,11,12,13,14]. Moreover, inadequate hygiene and waste disposal practices in food processing, coupled with substandard manufacturing methods, can strongly increase the risk of viral contamination in foods [15,16,17,18,19].

In the food industry, different sanitizing technologies are applied such as the use of radiation, heat, different chemicals, gaseous ozone [20], gaseous chlorine dioxide [21], and high hydrostatic pressure [22]. Nevertheless, these solutions are often effective only for a limited time and present a lot of disadvantages, encouraging the exploration of innovative and alternative approaches to improve cleaning procedures and food safety. Indeed, in recent years, several studies have been concentrated on less toxic sanitizing solutions, involving the identification of new natural compounds to develop sustainable and efficient cleaning procedures for food processing, transformation, and storage surfaces able to prevent the initial microbial attachment and eliminate the microorganisms from the food contact materials.

Recently, antimicrobial peptides (AMPs), also known as host-defence peptides, have attracted great attention [17]. AMPs are natural molecules present in the innate immune system of almost all living organisms, invertebrates, and vertebrates, identified as potential agents with therapeutic effects as they exhibit marked antibacterial, antiviral, antiparasitic, and antifungal properties. Generally, AMPs are derived from different microorganisms, such as mammals, plants, arthropods, reptiles, and amphibians [16,23,24,25] or are synthetically generated. AMPs represent a heterogeneous class that shares several main physicochemical characteristics: (i) the presence of basic amino acids conferring a net positive charge; (ii) the presence of 50% hydrophobic amino acids; and (iii) the ability to assume alpha-helix or beta-sheet conformations during the interaction with the target cell membrane. To date, more than 3000 AMPs have been officially classified and registered in the AMP database, with over 300 discovered in frog skin [26,27].

Previous studies have reported that antiviral AMPs target various stages in the viral lifecycle, especially attachment, entry, and fusion [28,29]. Attention has been directed toward identifying peptides targeting viral proteins [28]. AMPs can also neutralize both RNA and DNA viruses by incorporating them into the viral envelope [30,31], causing membrane instability and disruption and preventing host cell infections [32,33]. AMPs such as defensins can also decrease the binding of viruses to host cells by interacting with viral glycoproteins and inhibiting the attachment of herpes simplex viruses (HSV) to the surface of host cells [34]. Additionally, some antiviral AMPs can prevent the entry of the viral particles into the host cells by binding to specific mammalian receptors [35,36], while others can cross the cell membrane, prompting changes in the gene expression profile of the host cells and stimulating their defence system against viruses [37,38].

Temporins, derived from *Rana temporaria*, are peptides consisting of 10–13 amino acid residues with a slight cationic charge. In detail, their action is principally focused on enveloped viruses, including HSV-1 [39,40] and influenza A [17], although the mechanism of action is different: (i) some can act at the intracellular level by destroying the viral envelope and/or preventing the virus–membrane host cell fusion (influenza virus, HSV-1); or (ii) others can interfere in viral replication by preventing the release of new virions (parainfluenza viruses) [17,18].

Compared to the enveloped viruses, the absence of a lipid envelope in the naked variants may affect the antiviral mechanisms and the effectiveness of antiviral compounds. This may explain why there are few reports on the action of AMPs against non-enveloped viruses. In a previous study, the antiviral effects of cathelicidin-derived peptides were evaluated against non-enveloped Enterovirus 71 (EV71) with a single-stranded positive-sense RNA, belonging to the Picornaviridae family [38]. Some of these compounds have already shown antiviral activity against naked viruses in vitro, such as human rhinovirus and adenovirus [38]. In this case, it was demonstrated that the mechanisms of action can be also more complex and can involve different steps of the infection.

We have recently demonstrated the antibacterial activity of a panel of designed AMPs against food pathogen contaminants, including Gram-positive and Gram-negative bacteria and fungi but not viruses [41,42,43,44,45]. The structural properties and mechanism of action of these peptides have been elucidated previously and these compounds were also found to be non-cytotoxic [41,42,43,44,45].

The objective of the present study was to evaluate the virucidal effect of the same antimicrobial peptides that were previously evaluated against bacteria on two non-enveloped foodborne viruses, MNV-1 and HAV. Moreover, to expand the panel of peptides, two additional compounds were included in the tests against the viruses, the newly designed RiLK30 and the already known HIV inhibitor AVP2 [46], which were structurally characterized. Attempts were also undertaken to elucidate the mechanism of action of two of these peptides.

Hence, the present work aims to consolidate the current knowledge and findings on the antiviral properties of antimicrobial peptides and their potential applications.

## 2. Results and Discussion

### 2.1. Peptide Design

Due to the lack of drugs for the treatment of many viral infections and the increase in multidrug-resistant viruses, there is a high urgency to develop new compounds to fight these pathogens [47]. Recently, different studies have shown that antimicrobial peptides (AMPs) can also exert antiviral effects in addition to the well-known anti-bacterial or anti-fungal activities [38,48,49], thus making them ideal candidates for the development of antiviral drugs.

In our previous studies, the structural characterization, as well as the antibacterial and antifungal activities of the peptides 1018-K6 [50], MTP1, RiLK1 [51], and RiLK3, were demonstrated [42,43,44,45,46], thus leading to the current study’s investigations of their possible antiviral effects that have not been studied so far, considering that the ultimate goal of the research is to find agents that exhibit a broad-spectrum antimicrobial activity and are therefore attractive for manufacturing applications. Moreover, two further peptides were considered in our study to expand the panel of potential antiviral compounds (Table 1), the newly designed RiLK30 and the already known HIV inhibitor AVP2 [46], which was used as a reference. Concerning RiLK30, it was selected among a series of RiLK1-derivatives that were rationally designed by following specific and systematic amino acid substitutions in the parental RiLK1 sequence that, based on our own experience, it is crucial to preserve certain structural features, like the balance between the positive charges, hydrophobicity, and content of lipophilic residues such as Trp, that promote the peptide–microbial membrane interactions and maintain antimicrobial activity [52].

It is worth noting that RiLK1 was chosen as the lead compound among the other peptides available because of its broad spectrum of antimicrobial activity and its rapid production at low cost, being a smaller peptide than 1018-K6 and MTP-1, thus representing the best candidate to accelerate further studies and develop new molecules. The reliability of the new set of analogues in terms of antiviral activity was predicted using Meta-iAVP software [53], and based on this analysis, the RiLK30 variant, where all the arginine (R) residues within the RiLK1 sequence were exchanged with lysine (K) and the single Lys at position 3 with Arg, was selected for the in vitro antimicrobial tests. The physico-chemical characteristics of all the peptides under investigation predicted using the available software applications PlifePred, APD3, and Meta-iAVP [53,54,55] are reported in Table 1.

### 2.2. Structural Characterization of RiLK30 and AVP2

As for the antibacterial activity, the interaction of AMPs with membrane components frequently correlates with antiviral effects, suggesting that the presence of a membrane could be one of the essential conditions in inducing the peptide conformation capable of inhibiting viral infection [56,57,58].

In this context, to acquire important information on how the conformational properties of peptides might influence the structure–function relationships and their distinct membrane interaction profiles, the propensity of the new peptides, RiLK30 and AVP2, to adopt a defined secondary structure and to undergo conformational changes were examined by circular dichroism and fluorescence spectroscopy. These analyses were performed in the presence of a negatively charged SDS that is commonly used as a biomimetic membrane model to study the structural features of membranotropic molecules. In previous studies, the structure and the conformational stability of 1018-K6, MTP1, RiLK1, and RiLK3 have been already elucidated in detail [41,42,43,44,45]. Briefly, all these four peptides exhibited high structural stability across a wide range of pH (2–11) and temperature (4–90 °C), but they assumed different conformations in mimicked membrane solutions such as mixed α-helix/β-sheet for MTP1, β-sheet for 1018-K6 and higher-ordered self-aggregates, assembling into bigger oligomeric species in equilibrium with partially structured monomers for RiLK1 and RiLK3.

**Figure 1:**
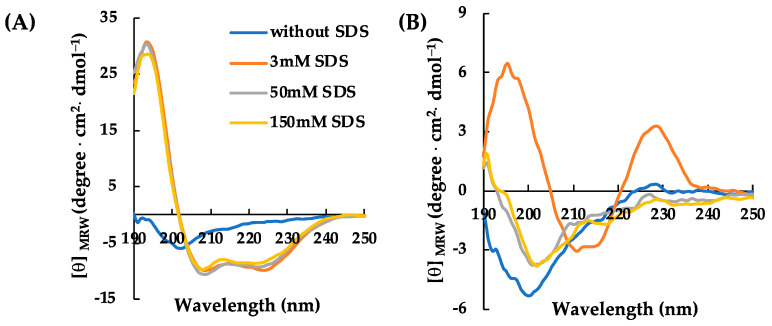
Effect of SDS concentration on the secondary structure of AVP2 and RiLK30 monitored by circular dichroism. Far-UV CD spectra of (**A**) AVP2 and (**B**) RiLK30 at 50 µM concentration and 25 °C in 10 mM Tris-HCl buffer, pH 7.0, in the absence (blue lines) or presence of SDS at different concentrations.

Concerning RiLK30 and AVP2, in the first set of experiments the CD spectra were recorded at a fixed peptide concentration (50 μM) in 10 mM Tris-HCl buffer (pH 7.0) and in the absence or presence of SDS solutions, below and above the critical micelle concentration. As shown in Figure 1, both peptides present a dichroic spectrum in an aqueous solution with the typical shape of a random coil structure characterized by a negative band at ~200 nm. Instead, the presence of SDS significantly affects the CD spectrum of the peptides that underwent a variation in shape when in contact with the oppositely charged amphiphile. Specifically, AVP2 adopts a typical α-helical conformation in SDS solutions at concentrations both lower (3 mM) and higher (50 and 150 mM) than the micellar one (Figure 1A). Conversely, RiLK30 shows a more complex conformation that is not correlated with the common secondary structure elements (α-helix, β-strand, or random coil) (Figure 1B) and can be associated with its high propensity to self-aggregate, as already observed for its analogs RiLK1 and RiLK3 [41,42,43].

**Figure 2:**
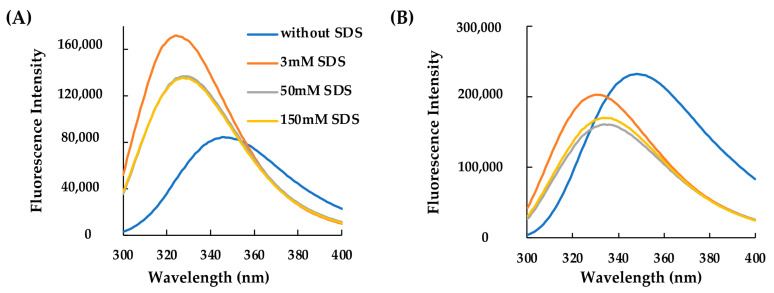
Effect of SDS concentration on the secondary structure of AVP2 and RiLK30 monitored by fluorescence spectroscopy. Fluorescence spectra of (**A**) AVP2 and (**B**) RiLK30 at 50 µM concentration and 25 °C in 10 mM Tris-HCl buffer, pH 7.0, in the absence (blue lines) or presence of SDS at different concentrations.

These results were also confirmed by fluorescence spectroscopy analyses, which demonstrated the structuring of the two peptides induced by the microenvironment surrounding the tryptophan which became more hydrophobic following the addition of the detergent at different concentrations (Figure 2A,B).

**Figure 3:**
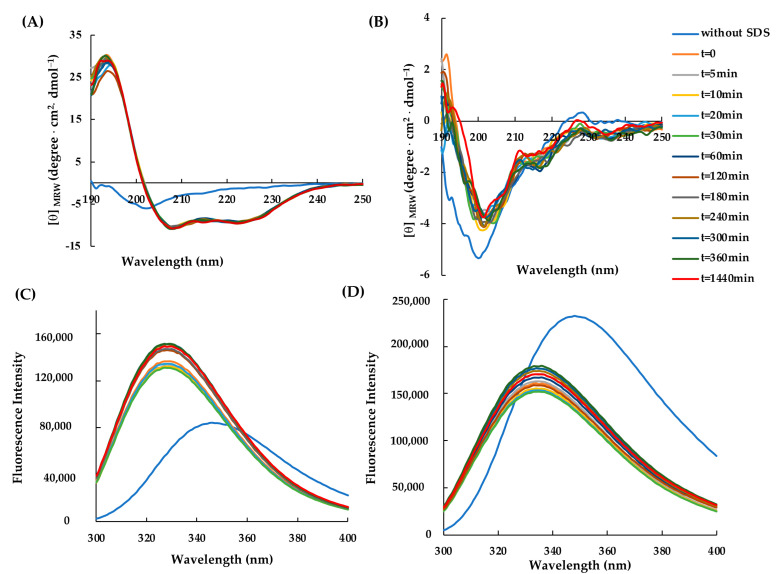
Time-dependent effect of SDS on the secondary and tertiary structure of AVP2 and RiLK30 monitored by spectroscopic techniques. Far-UV circular dichroism spectra of (**A**) AVP2 and (**B**) RiLK30. Intrinsic fluorescence emission spectra of (**C**) AVP2 and (**D**) RiLK30. All spectra were recorded at a peptide concentration of 50 µM in 10 mM Tris-HCl, pH 7.0, in the presence or absence (blue lines) of SDS (50 mM) up to 24 h incubation at 25 °C.

Subsequently, to study the conformational rearrangements of RiLK30 and AVP2 over time, the molecules were incubated for 24 h at 37 °C and pH 7.0, after which the dichroic (Figure 3A,B) and fluorescence (Figure 3C,D) spectra were acquired in the presence of SDS 50 mM. These analyses highlighted that the peptides did not appear to undergo significant changes in the following 24 h once their conformational distribution had been assumed (t = 0).

**Figure 4:**
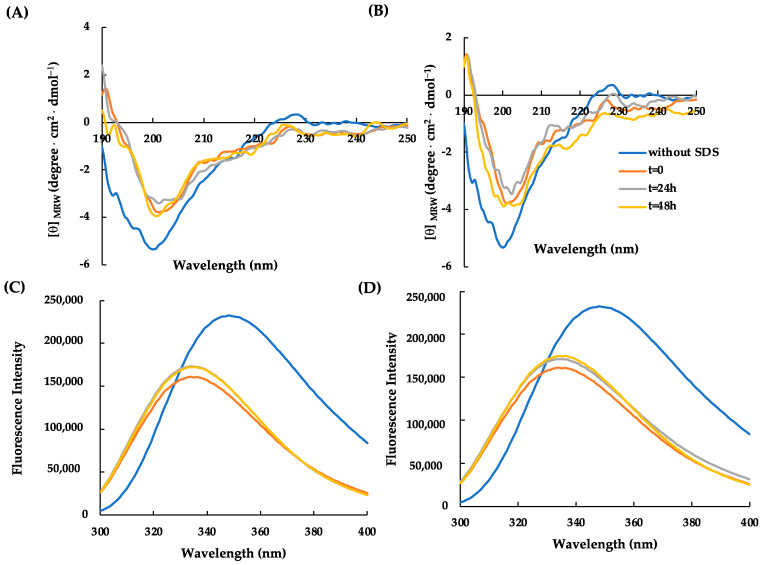
Effect of temperature on the secondary and tertiary structure of RiLK30 monitored by spectroscopic techniques. Far-UV CD spectra of RiLK30 at (**A**) 4 °C and (**B**) 25 °C. Intrinsic fluorescence emission spectra of RiLK30 at (**C**) 4 °C and (**D**) 25 °C. All spectra were recorded at a peptide concentration of 50 µM in 10 mM Tris-HCl buffer, pH 7.0, in the presence or absence (blue lines) of SDS (50 mM) up to 48 h incubation at 25 °C.

**Figure 5:**
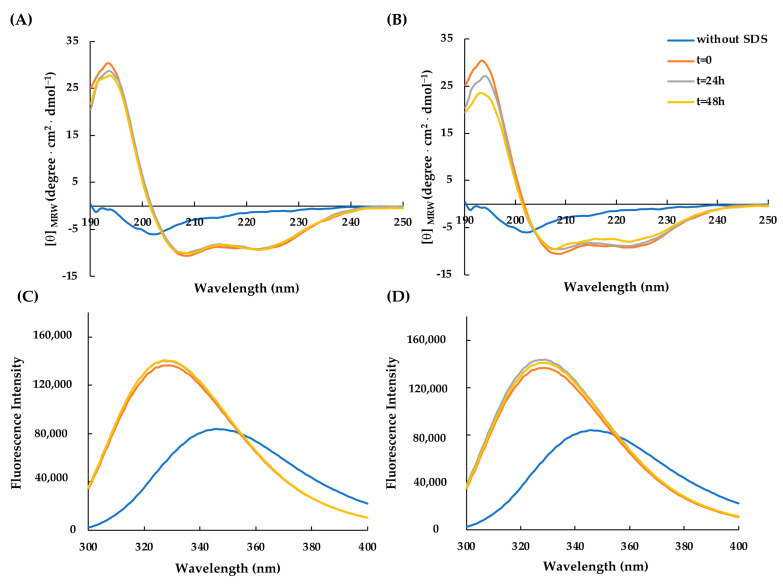
Effect of temperature on the secondary and tertiary structure of AVP2 monitored by spectroscopic techniques. Far-UV CD spectra of AVP2 at (**A**) 4 °C and (**B**) 25 °C. Intrinsic fluorescence emission spectra of AVP2 at (**C**) 4 °C and (**D**) 25 °C. All spectra were recorded at a peptide concentration of 50 µM in 10 mM Tris-HCl buffer, pH 7.0, in the presence or absence (blue lines) of SDS (50 mM) up to 48 h incubation at 25 °C.

It has been pointed out that the antimicrobial activity of some AMPs is greatly weakened by some physical parameters, such as temperature and salt. Therefore, the conformational and structural stability of RiLK30 (Figure 4) and AVP2 (Figure 5) in response to different temperature conditions were verified by incubating the peptides for 48 h at pH 7.0 and at 4 °C or 25 °C. The results obtained following spectroscopic analyses revealed that the thermal treatment is not able to induce conformational changes in both peptides, confirming their high thermal stability for at least 48 h in SDS micellar solution.

### 2.3. Antiviral Activity of Peptides

According to the Antimicrobial Peptide Database (APD), antiviral effects have been reported for only 185 out of more than 3000 registered AMPs due to the more difficult evaluation of antiviral effects [55]. Thus, considering that the issue of emerging and re-emerging infectious diseases, especially those related to viruses, has become an increasingly important area of concern in public health, the discovery of novel antiviral compounds deserves additional effort.

In this context, our peptides were tested against norovirus (NoV) and hepatitis A virus (HAV), which are considered two of the principal enteric viruses causing food and waterborne outbreaks due to their ability to cause infection in very low doses and to survive in an external environment for a long time. NoVs and HAV are small non-enveloped viruses with a positive-sense, single-stranded RNA genome. For NoV, the murine species (strain MNV-1) was used in the viability studies due to the lack of in vitro cultivation methods for human norovirus (HuNoV). Indeed, MNV-1 is currently recognized as the most widely accepted surrogate model, being the most closely related virus to HuNoV in size, capsid structure, genomic organization, and replication cycle [59,60].

The first step of our analysis was devoted to discriminating between cytotoxicity and virucidal effects. Therefore, to exclude the possibility that our peptides could be cytotoxic on RAW 264.7 or Frp3 cells, the host cell lines used to assess the antiviral activity against MNV-1 and HAV, different concentrations of each peptide ranging from 10 to 100 μM were tested by MTT assays. The results demonstrated no cytotoxicity in all the tested ranges (data not shown). Subsequently, 40 and 80 µM were the concentrations chosen to perform the next experiments, and the potential virucidal activity of the peptides against HAV and MNV-1 was assessed at two different temperatures.

In a previous work, the antiviral effects of RiLK1 and RiLK3 were already investigated against hepatitis A virus (HAV) following treatment at room temperature [45], and the obtained results are reported in Table 2. Briefly, RiLK1 was able to reduce HAV infectivity by more than 1 log in comparison with the untreated virus, displaying a log reduction value (LRV) of 1.4 at 80 μM and 1.1 at 40 μM concentration [45]. Conversely, RiLK3 was unable to inhibit HAV infection in the same concentrations tested. Therefore, based on these preliminary results, further insights into the virucidal activity of both RiLK1/RilK3 and the other peptides were provided in this study (Table 2 and Table 3). Concerning RiLK1 and RiLK3, the results obtained for HAV at 4 °C were comparable to those previously obtained at room temperature [45] (Table 2).

As regards MNV-1 (Table 3), a virucidal effect was observed for RiLK1 after treatment at room temperature (LRV of 1.2 and 1.0 at 80 µM and 40 µM, respectively), while RiLK3 was not effective regardless of the concentration or temperature condition applied. RiLK30 showed no virucidal activity in any of the conditions tested, neither toward HAV nor MNV-1 (Table 3).

On the other hand, 1018-K6 and MTP1 proved to be effective on HAV at 40 µM and 80 µM and at the two temperatures, causing an infectivity reduction of 1.3–1.4 log for 1018-K6 and 0.9 to 1.5 log for MTP-1. However, no significant effects were exhibited by these peptides toward MNV-1. Conversely, AVP2 was active against MNV-1 at both temperatures (LRV of 0.9 and 1.0 log) but provided a limited reduction in infectivity on HAV.

In terms of the reduction in viral infectivity, HAV infectious titer was decreased up to 96.7% (1.4 LRV) by RiLK1 and 1018-K6, and up to 97.3% (1.5 LRV) by MTP1. Instead, MNV-1 infectivity was reduced up to 94.1% (1.2 LRV) by RiLK1 and up to 91.3% (1.0 LRV) by AVP2. Therefore, only RiLK1 showed effectiveness toward both viruses.

Based on these in vitro results, RiLK1 was chosen as a model molecule for an in-depth analysis to elucidate the mechanism of its antiviral action in comparison with the its analog RiLK3, differing by only one amino acid, to understand the significant changes in virucidal activity observed between the two peptides. To this aim, four types of assays, varying in the timing of the addition of the drug, were performed as described in Section 3.8 (virus pre-treatment assay, cell pre-treatment assay, attachment assay, and entry assay). The results are reported in Table 4 for HAV and in Table 5 for MNV-1.

Consistent with the previous results, RiLK1 displayed a reduction in the infectious titer of 1.4 log for HAV and 1.2 log for MNV-1 in the virus pre-treatment assay through direct interaction with viral particles. Similarly, an LRV of 1.4 for both HAV and MNV-1 was shown in the attachment assay through the inhibition of virus attachment to the cell’s receptors.

Furthermore, RilLK1 also exhibited activity in preventing the entry of MNV-1 into the cells (entry assay, LRV 1.0). On the other hand, RiLK3 revealed a more pronounced antiviral action through the interaction with the cells (cell pre-treatment assay, LRV 0.9 and 1.0 for HAV and MNV-1, respectively) and not directly on the virus. Moreover, RiLK3 also affected the viral attachment to cells (LRV 1.0 for both HAV and MNV-1) and inhibited MNV-1 entry in the host cells (Table 5).

Considering that RiLK3 derives from RiLK1 by replacing a single lysine residue with arginine, it is important to underline how small variations in the peptide structure can determine significant changes in their antiviral properties, influencing the mechanism of action. In this case, the single amino acid substitution affects the interaction of RiLK3 with the virus particles, but it does not prevent the infectious process.

The obtained results are particularly interesting as the use of peptides against non-enveloped viruses is limited as most AMPs act during the viral fusion to the cell membrane, exclusive of enveloped viruses. Therefore, there is still more work to be conducted in the design of inactivators against this class of pathogens because the structure and function of the receptor-binding domains in their capsid proteins have not been completely elucidated [61].

### 2.4. Antibacterial Activity of Peptides

The zoonoses that occur most frequently in the industrialized world today are foodborne infections caused by bacteria enzootic to food animals such as *Campylobacter* and *Salmonella*. As a result, campylobacteriosis and salmonellosis have emerged as critical global health problems with a high socioeconomic burden. Hence, it is important to identify novel ‘weapons’ to reduce and/or prevent these infections.

**Figure 6:**
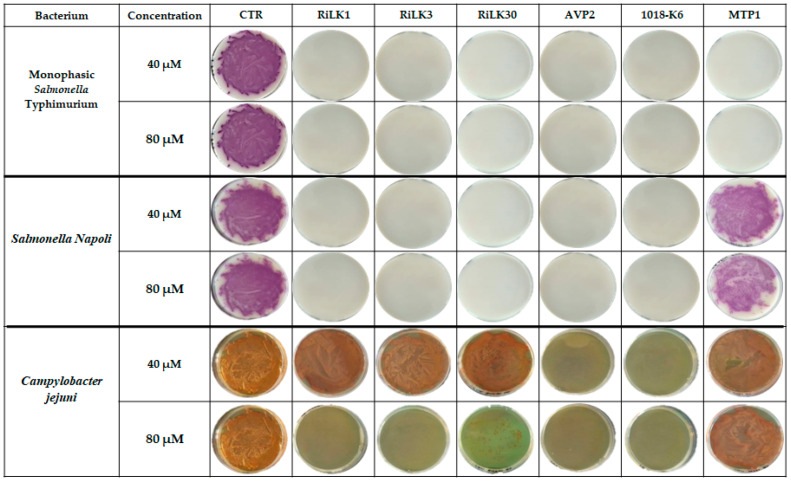
Antibacterial activity of our peptides against the foodborne pathogens monofasic *Salmonella* Typhimurium, *Salmonella* Napoli, and *Campylobacter jejuni*. CTRL: microorganism without treatment. Bacterial cultures treated, or not, with peptides at two different concentrations were seeded on selective plates. The images are representative of three independent experiments performed in triplicate.

In this study, all the designed peptides were tested against *Campylobacter jejuni*, the monophasic variant of *S*. Typhimurium (MVST), and *Salmonella* Napoli, three of the most relevant zoonotic pathogens reported to be strongly involved in human infections in the EU. As shown in Figure 6, all peptides exhibited strong bactericidal activity toward both serovars of *Salmonella*, being able to completely inhibit the growth of bacteria already at 40 μM concentration except for MTP-1 that exerted no detectable killing effects against *Salmonella* Napoli, even at the highest concentration tested (80 μM). Concerning *Campylobacter jejuni* strains, only AVP2 and 1018-K6 presented 100% inhibition already at a concentration of 40 µM while the RiLK-group was active only at 80 µM concentration, with RiLK30 being the most ineffective. By contrast, MTP-1 did not display any inhibitory activity at both concentrations.

These findings prompt interesting future applications of these peptides in a mixture exploiting the high potential of their synergistic action to treat various foodborne microbial infections.

## 3. Materials and Methods

### 3.1. Virus Strains and Cell Lines

Murine norovirus strain 1 (MNV-1) was used as a surrogate for human norovirus (NoV). The virus was replicated in RAW 264.7 cells (murine macrophage cell line) cultured in Dulbecco’s modified Eagle’s medium (DMEM) supplemented with 1% glutamine, 1% non-essential amino acids, and 2% fetal bovine serum (FBS). Hepatitis A virus (HAV) strain HM-175 was replicated in Frp3 cells (non-human primate cell line derived from fetal kidney) cultured in minimum essential medium (MEM) with Earle’s salts supplemented with 1% glutamine, 2% non-essential amino acids, and 2% FBS. Both viruses were incubated in 5% CO_2_ at 37 °C. All cell culture media were provided by EuroClone (Milan, Italy). The viral suspensions were prepared by freeze and thaw lysis (1 cycle for MNV-1 and 3 cycles for HAV) of infected monolayers, clarified using low-speed centrifugation (800× *g*) to remove residual cell debris, then divided in aliquots, and stored at −80 °C until use. The obtained HAV and MNV-1 stock suspensions had a final concentration of 4.7 ± 0.2 log TCID_50_/mL and 4.5 ± 0.2 log TCID_50_/mL, respectively, calculated by determining the 50% tissue culture infectious dose with the Reed and Muench method [62], using tenfold serial dilutions in 24-well plates.

### 3.2. Antimicrobial Peptides

The antimicrobial peptides (AMPs) tested in the study were RiLK1, RiLK3, RiLK30, 1018-K6, MTP1, and AVP2. The peptides were purchased from GenScript Biotech (Leiden, The Netherlands) and obtained at >95% purity. The peptides were stored as a lyophilized powder at −20 °C until use. The peptides were resuspended in 5 mM DMSO to prepare the peptide stock solutions (500 µM) and stored at −20 °C until use. The following web server and software were used for determining the main physicochemical properties of the peptides: PlifePred (PPred) [54], Antimicrobial Peptide Database3 (APD3) [55], and Meta-iAVP [53].

### 3.3. Circular Dichroism (CD) Spectroscopy

Circular Dichroism (CD) analysis was performed by a Jasco J-810 spectropolarimeter. The samples were loaded into a quartz cuvette of 0.1 cm path length (Hellma Analytics), and the spectra were recorded in the 195–250 nm range at a scan speed of 20 nm/min, averaging 5 scans. CD experiments were carried out in 10 mM Tris-HCl buffer, pH 7.0, as a function of SDS concentrations (3, 50, and 150 mM) at a peptide concentration of 50 µM. The folding kinetic measurements of the peptides were performed after the addition of SDS (50 mM) to each sample (50 µM in 10 mM Tris-HCl, pH 7.0) up to 24 h of incubation. For thermal stability analyses, the peptides were prepared to a final concentration of 50 μM in 10 mM Tris-HCl, pH 7.0, and then they were incubated at 4 and 25 °C up to 48 h before acquiring the CD spectra in the presence of 50 mM SDS. A blank spectrum of a sample containing all components except the peptide was acquired for the baseline correction of the CD spectra of the peptide. The mean residue ellipticity ([θ], deg cm^2^ dmol^−1^) was obtained by the equation [θ] = 100 θ/cnl, where θ is the ellipticity (mdeg), c is the peptide concentration (mM), n is the number of residues, and l is the path length (cm).

### 3.4. Fluorescence Spectroscopy

Tryptophan (Trp) fluorescence emission spectra were recorded at 25 °C on a Shimadzu RF-6000 spectrofluorometer (Kyoto, Japan), with both excitation and emission slit widths set at 5 nm. The intrinsic Trp was excited at a wavelength of 280 nm and the emission was monitored between 300 and 400 nm. Fluorescence measurements were carried out in 10 mM Tris-HCl buffer, pH 7.0, as a function of SDS concentrations (3, 50, and 150 mM) at a peptide concentration of 50 µM. The folding kinetic experiments of AVP2 and RiLK30 were performed after the addition of SDS (50 mM) to each sample (50 µM concentration in 10 mM Tris-HCl buffer, pH 7.0) up to 24 h of incubation. The effect of temperature on peptide folding was analyzed by dissolving the peptides at a final concentration of 50 µM in 10 mM Tris-HCl buffer, pH 7.0, and incubating them at 4 and 25 °C up to 48 h. The spectra were recorded in the presence of 50 mM SDS.

### 3.5. Cytotoxicity Determination of Peptides on Cells

To identify the peptide concentrations that had no cytotoxic effect, preliminary tests were performed on cell cultures. Cell viability was evaluated by the 3-(4,5-dimethilthiazol-2-yl)-2,5-diphenyl tetrazolium bromide (MTT) assay [63]. Peptide solutions were prepared in serum-free MEM at different concentrations in the range from 10 to 100 µM (10, 20, 40, 80, 100 µM), treated overnight at 4 °C with antibiotic/antimycotic solution (Euroclone) and 100 µL were assayed on 24–48 h cell monolayers in a 24-well plate. The monolayers added with the peptides were incubated for 1 h at 37 °C in 5% CO_2_. Thereafter, cells were washed twice with Dulbecco’s phosphate buffer solution (DPBS, EuroClone) and maintained in culture with DMEM/MEM supplemented with 2% of FBS for 48 h in 5% CO_2_ at 37 °C. After that, the medium was removed and 300 µL of MTT (Sigma Aldrich, Milan, Italy) solution (5 mg/mL) was added. The monolayers were incubated for 15–30 min at 37 °C in 5% CO_2_, the MTT was removed, and 500 µL DMSO was added to each well to dissolve the purple formazan crystals. The absorbance was measured at 570 nm. DMSO and culture medium were used as controls.

### 3.6. Virucidal Effect of Peptides

Solutions 40 µM and 80 µM of each peptide of the panel (RiLK1, RiLK3, RiLK30, 1018-K6, MTP1, and AVP2) were chosen for the virucidal tests. HAV and MNV-1 were assayed at a concentration of 4.7 ± 0.2 log TCID_50_/mL and 4.5 ± 0.2 log TCID_50_/mL, respectively. The suspensions containing the peptides and viruses were incubated for 1 h at room temperature (RT,20 ± 2 °C) and 4 °C. These temperatures were chosen to simulate the use of sanitizing solutions in food production/processing environments. Residual viral infectivity was evaluated by titration (TCID_50_/mL) on Frp3 cells for HAV and on RAW 264.7 cells for MNV-1. Untreated HAV and MNV-1 suspensions and 40 µM and 80 µM AMP solutions, incubated at the same conditions, were used as controls. Each treatment condition was assayed in triplicate. For titration, 100 µL of serial tenfold dilutions of each sample were assayed in 24-well tissue culture plates containing 24–48 h cell monolayers and incubated for 1 h in 5% CO_2_ at 37 °C. After that, the wells were washed twice with 200 µL of PBS, and 500 µL of DMEM/MEM, supplemented with 2% of FBS, were was added to each well; MNV-1 and HAV incubations were carried for up to 6 days and 14 days, respectively, in 5% CO_2_ at 37 °C with daily visual inspection to determine the cytopathic effect. The virucidal efficacy of AMPs was estimated by comparing the titers of treated and untreated viral suspensions.

### 3.7. Antibacterial Assays

The monophasic variant of *Salmonella* Typhimurium, *Salmonella* Napoli, and *Campylobacter jejuni* were isolated by the Laboratory of Microbiological Food Control—Department of Food Microbiology of the Istituto Zooprofilattico Sperimentale del Mezzogiorno in Portici (Naples, Italy) in raw and processed foodstuffs of animal origin. Specifically, the monophasic variant of *S*. Typhimurium and *S*. Napoli were isolated from bivalve mollusks, while *C. jejuni* was isolated from poultry meat. Bacterial cells were cultured at 37 °C in the appropriate culture media (*S*. Typhimurium and *S*. Napoli in buffered peptone water (Thermo Fisher, Milan, Italy) and *C. jejuni* in Bolton Broth (Oxoid, Madrid, Spain)) until collection and then diluted in fresh broth to a final concentration of 1.5 × 10^3^ colony forming units (CFUs)/mL. Thereafter, peptide stock solutions in DMSO were added to the bacterial suspension at the same concentrations used in the virucidal assays (40 and 80 µM) and incubated at 37 °C for 6 h. Samples containing only cell suspension and DMSO were used as controls. Therefore, 50 μL of each bacterial cell suspension was transferred onto selective agar plates (*S*. Typhimurium and *S*. Napoli, Salmonella chromogenic agar—Oxoid, Madrid, Spain—and *C. jejuni*, modified Charcoal Cefoperazone Deoxycholate Agar (Liofilchem, Roseto degli Abruzzi, Italy)) in microaerophilic conditions and incubated for 24 h at 37 °C for *S*. Typhimurium and *S*. Napoli (ISO) 6579-1:2020) and 48 h at 41 °C for *C. jejuni* (ISO 10272-2:2017). All values were obtained as the mean of three independent experiments conducted in triplicate.

### 3.8. Mechanism of Action of RiLK1 and RiLK3 Peptides

Four sets of experiments were performed to suppose the effect of RiLK1 and RiLK3 on HAV and MNV-1 [29]. To this aim, 80 µM solutions of the two peptides and HAV and MNV-1 suspensions at a final concentration of 4.7 ± 0.2 log TCID_50_/mL and 4.5 ± 0.2 log TCID_50_/mL, respectively, were used in the following experiments:

i) A virus pre-treatment assay to assess the direct interaction between the virus and peptide: the incubation of the peptide with viral suspension for 1 h at room temperature was carried out, followed by its incubation on cells for 1 h at 37 °C. Then, cell monolayers were washed twice with PBS and incubated with 500 µL DMEM/MEM supplemented with 2% of FBS to determine the TCID_50_/mL.
ii) A cell pre-treatment assay to assess the interaction between the peptide and cells before the viral infection: cell monolayers were preincubated with 100 µL of peptide solution for 1 h at 37 °C, followed by the removal of the peptide, addition to cells of viral suspensions for 1 h at 37 °C, washing step, and culture with 2% FBS DMEM/MEM.
iii) An attachment assay to assess the possible action of the peptide at the stage of virus attachment to cells: cell monolayers were incubated with 100 µL of a suspension containing both the peptide and virus and were incubated for 1 h at 4 °C. Subsequently, the cell monolayers were washed with PBS and incubated for 1 h at 37 °C with a culture medium followed by a washing step and the addition of culture with 2% FBS DMEM/MEM.
iv) An entry assay to assess the possible action of the peptide at the stage of virus internalization into cells: cell monolayers were incubated with 100 µL of the viral suspension for 1 h at 4 °C, followed by the addition of the peptide and incubation for 1 h at 37 °C. Cells were then washed with PBS, and 2% FBS DMEM/MEM was finally added.

Untreated viral suspensions and peptide solutions, incubated at the same conditions, were used as controls.

### 3.9. Statistical Analysis

For each treatment, the average and standard deviation of the triplicate analyses were calculated. The virucidal effects of AMPs was assessed by calculating the log reduction value (LRV), i.e., the difference between the infectious titer (log-transformed) of untreated versus treated samples. The statistical significance of differences between treated and untreated samples was determined through one-way analysis of variance (ANOVA) with Bonferroni post hoc comparisons, with a significance level of *p* < 0.05 (GraphPad Prism v9.5.0, software San Diego, CA, USA).

## 4. Conclusions

The lack of therapeutic agents against many important viral infections along with the ever-growing problematic of drug resistance constitutes a public health emergency of international concern, requiring the urgent discovery of novel antimicrobial molecules, possibly with low toxicity and limited costs. In this scenario, the natural compounds AMPs might provide a solution for preventing and controlling foodborne diseases. To date, few studies have been performed to investigate how AMPs interact with viruses and how such interaction affects viral infectivity [29,38,64].

In the present study, the antiviral activity of six synthetic peptides with different structural features was evaluated for the first time against two non-enveloped enteric viruses, HAV and MNV-1, a surrogate for human norovirus, together with the mechanism of action of the two of them. Our results demonstrated that RiLK1 was the most promising peptide among those tested, showing a remarkable virucidal effect against both viruses by interacting directly with the viral particles or inhibiting the viral bonding to the cell receptors. In this regard, it can be supposed that the antiviral mechanism was not triggered by the peptide conformation, as evidenced by the results obtained by AVP2 that was not able to explicate a virucidal effect on both MNV-1 and HAV viruses, although exhibiting the typical α-helical structure.

These findings, together with those previously obtained against bacteria and fungi, could promote RiLK1 as a new promising ‘multifaceted’ agent with an attractive applicative potential due to its broad spectrum of antimicrobial activity. This peptide could be suitable as a natural surface and food sanitizer to prevent or reduce contamination by foodborne pathogens. However, further studies will be necessary to investigate the mechanism of action of all the other peptides to determine their antiviral activity and to evaluate a possible synergistic effect when used in a mixture.

## Figures and Tables

**Table 1:** Characteristics of the selected peptides.

Peptide	Sequence	MW (Da)	Net Charge	BI (kcal/mol)	Hydrophobicity	AVP ^§^
**RiLK1 ^a^**	RLKWVRIWRR-NH2 (10 aa)	1467.8	+6	4.70	−0.56	1
**RiLK3 ^b^**	RLRWVRIWRR-NH2 (10 aa)	1495.8	+6	5.6	−0.63	0.932
**RiLK30**	KLRWVKIWKK-NH2 (10 aa)	1383.8	+6	1.85	−0.36	0.994
**1018-K6 ^c^**	VRLIVKVRIWRR-NH2 (12 aa)	1594.0	+6	3.00	−0.35	0.558
**MTP1 ^d^**	KVSGVLFGTGLWVAL-NH2 (15 aa)	1545.8	+2	−1.68	0.23	0.744
**AVP2 ^e^**	GWFDVVKHIAKRF-NH2 (13 aa)	1601.9	+4	1.18	−0.10	0.926

All the peptides were C-terminally amidated. MW: molecular weight; BI: Boman index; ^a^ ref [44,45]; ^b^ ref [46]; ^c^ ref [43]; ^d^ ref [42]; ^e^ ref [47]; ^§^ antiviral prediction.

**Table 2:** In vitro effect of peptides on HAV.

	Viral Titre after Treatment (logTCID_50_/mL ± SD)		LRV (logTCID_50_/mL ± SD)	
Treatment	4 °C	RT	4 °C	RT
Untreated HAV	4.7 ± 0.2	4.7 ± 0.2 ^a^	n.r.	n.r. ^a^
RiLK1 [80 µM]	3.6 ± 0.1 *	3.3 ± 0.2 *^,a^	1.1 ± 0.3 *	1.4 ± 0.4 *^,a^
RiLK1 [40 µM]	3.9 ± 0.2	3.6 ± 0.1 *^,a^	0.8 ± 0.4	1.1 ± 0.3 *^,a^
RiLK3 [80 µM]	4.6 ± 0.1	4.4 ± 0.1 ^a^	0.1 ± 0.3	0.3 ± 0.3 ^a^
RiLK3 [40 µM]	4.6 ± 0.3	4.4 ± 0.1 ^a^	0.1 ± 0.2	0.3 ± 0.3 ^a^
RiLK30 [80 µM]	4.3 ± 0.2	4.1 ± 0.2	0.4 ± 0.4	0.6 ± 0.4
RiLK30 [40 µM]	4.3 ± 0.2	4.1 ± 0.2	0.4 ± 0.4	0.6 ± 0.4
1018-K6 [80 µM]	3.3 ± 0.2 *	3.3 ± 0.2 *	1.4 ± 0.4 *	1.4 ± 0.4 *
1018-K6 [40 µM]	3.4 ± 0.1 *	3.3 ± 0.2 *	1.3 ± 0.3 *	1.4 ± 0.4 *
MTP1 [80 µM]	3.8 ± 0.2 *	3.5 ± 0.1 *	0.9 ± 0.4 *	1.2 ± 0.3 *
MTP1 [40 µM]	3.6 ± 0.3 *	3.2 ± 0.2 *	1.1 ± 0.2 *	1.5 ± 0.4 *
AVP2 [80 µM]	4.3 ± 0.2	4.0 ± 0.3	0.4 ± 0.4	0.7 ± 0.5
AVP2 [40 µM]	4.3 ± 0.2	4.3 ± 0.2	0.4 ± 0.4	0.4 ± 0.4

n.r. = no reduction; LRV = log reduction value; RT = room temperature (20 ± 2 °C); * values significantly different from the untreated virus (LRV ≥ 0.5); ^a^ ref [46].

**Table 3:** In vitro effect of peptides on MNV-1.

	Viral Titre after Treatment (logTCID_50_/mL ± SD)		LRV (logTCID_50_/mL ± SD)	
Treatment	4 °C	RT	4 °C	RT
Untreated MNV-1	4.5 ± 0.2	4.5 ± 0.2	n.r.	n.r.
RiLK1 [80 µM]	4.3 ± 0.2	3.3 ± 0.2 *	0.2 ± 0.4	1.2 ± 0.4 *
RiLK1 [40 µM]	4.3 ± 0.2	3.5 ± 0.2 *	0.2 ± 0.4	1.0 ± 0.4 *
RiLK3 [80 µM]	4.5 ± 0.2	4.5 ± 0.1	0 ± 0.4	0 ± 0.3
RiLK3 [40 µM]	4.5 ± 0.1	4.5 ± 0.2	0 ± 0.3	0 ± 0.4
RiLK30 [80 µM]	3.9 ± 0.2	4.5 ± 0.1	0.6 ± 0.4	0 ± 0.3
RiLK30 [40 µM]	4.3 ± 0.3	4.5 ± 0.2	0.2 ± 0.5	0 ± 0.4
1018-K6 [80 µM]	4.3 ± 0.2	4.3 ± 0.2	0.2 ± 0.4	0.2 ± 0.4
1018-K6 [40 µM]	4.5 ± 0.1	4.5 ± 0.2	0 ± 0.3	0 ± 0.4
MTP1 [80 µM]	4.5 ± 0.1	4.5 ± 0.2	0 ± 0.3	0 ± 0.4
MTP1 [40 µM]	4.4 ± 0.1	4.5 ± 0.2	0.1 ± 0.3	0 ± 0.4
AVP2 [80 µM]	3.6 ± 0.1 *	3.5 ± 0.1 *	0.9 ± 0.3 *	1.0 ± 0.3 *
AVP2 [40 µM]	3.6 ± 0.1 *	3.6 ± 0.1 *	0.9 ± 0.3 *	0.9 ± 0.3 *

n.r. = no reduction; LRV = log reduction value; RT = room temperature (20 ± 2 °C); * values significantly different from the untreated virus (LRV ≥ 0.5).

**Table 4:** Mechanism of action of RiLK1 and RiLK3 peptides on HAV infectivity.

	RiLK1		RiLK3	
	Viral Titre (logTCID_50_/mL ± SD)	LRV (logTCID_50_/mL ± SD)	Viral Titre (logTCID_50_/mL ± SD)	LRV (logTCID_50_/mL ± SD)
untreated virus	4.7 ± 0.2	-	4.7 ± 0.2	-
virus pre-treatment	3.3 ± 0.2 *	1.4 ± 0.4 *	4.4 ± 0.1	0.3 ± 0.3
cell pre-treatment	4.6 ± 0.1	0.1 ± 0.3	3.8 ± 0.2 *	0.9 ± 0.4 *
attachment	3.3 ± 0.2 *	1.4 ± 0.4 *	3.7 ± 0.2 *	1.0 ± 0.4 *
entry	4.6 ± 0.1	0.1 ± 0.3	4.2 ± 0.2	0.5 ± 0.4

LRV = log reduction value; * values significantly different from the untreated virus (LRV ≥ 0.5).

**Table 5:** Mechanism of action of RiLK1 and RiLK3 peptides on MNV-1 infectivity.

	RiLK1		RiLK3	
	Viral Titre (logTCID_50_/mL ± SD)	LRV (logTCID_50_/mL ± SD)	Viral Titre (logTCID_50_/mL ± SD)	LRV (logTCID_50_/mL ± SD)
untreated virus	4.5 ± 0.2	-	4.5 ± 0.2	-
virus pre-treatment	3.3 ± 0.2 *	1.2 ± 0.4 *	4.5 ± 0.1	0 ± 0.3
cell pre-treatment	4.2 ± 0.2	0.3 ± 0.4	3.5 ± 0.1 *	1.0 ± 0.3 *
attachment	3.1 ± 0.2 *	1.4 ± 0.4 *	3.5 ± 0.1 *	1.0 ± 0.3 *
entry	3.5 ± 0.2 *	1.0 ± 0.4 *	3.5 ± 0.1 *	1.0 ± 0.3 *

LRV = log reduction value; * values significantly different from the untreated virus (LRV ≥ 0.5).

## Data Availability

The data presented in the study are available in this manuscript.
